# Extubation-to-First Mobilisation Interval in Major Spine Surgery: A Prospective Study

**DOI:** 10.7759/cureus.114474

**Published:** 2026-08-13

**Authors:** Bharatkumar R Dave, Saurabh S Kulkarni, Shivanand C Mayi, Ajay Krishnan, Ravi Ranjan Rai, Mirant B Dave, Mikeson Panthackel, Arjit Vashishtha, Yogenkumar Adodariya, Rushikesh B Shahade, Kishan Panjwani

**Affiliations:** 1 Spine Surgery, Stavya Spine Hospital and Research Institute, Ahmedabad, IND; 2 Spine Surgery, Bhavnagar Institute of Medical Science (BIMS), Bhavnagar, IND; 3 Orthopaedics, King Edward Memorial Hospital and Seth Gordhandas Sunderdas (GS) Medical College, Mumbai, IND; 4 Orthopaedics, Lokmanya Tilak Municipal General Hospital and Lokmanya Tilak Municipal Medical College, Nagpur, IND; 5 Surgery, Government Medical College and General Hospital, Nagpur, Nagpur, IND

**Keywords:** early mobilisation, enhanced recovery after surgery (eras), extubation-to-mobilisation interval, major spine surgery, postoperative recovery

## Abstract

Background

Early mobilisation is a key component of Enhanced Recovery After Surgery (ERAS) protocols and has been associated with improved postoperative recovery following major spine surgery. However, limited prospective data exist regarding the impact of the interval between extubation and first mobilisation on postoperative outcomes.

Objective

This study aims to prospectively evaluate the extubation-to-first mobilisation interval in patients undergoing major spine surgery and assess its association with postoperative pain, analgesic requirements, complications, and hospital stay.

Methods

This prospective observational study was conducted at Stavya Spine Hospital and Research Institute, Ahmedabad. Adult patients undergoing planned major spine surgery, including multilevel fusion, instrumentation, and complex decompression procedures, were consecutively enrolled. The primary variable assessed was the interval (hours) between extubation and first out-of-bed mobilisation. Postoperative outcomes included serial Visual Analog Scale (VAS) pain scores, analgesic requirements, Oswestry Disability Index (ODI), complications, and duration of hospital stay.

Results

A total of 267 patients were included. The mean extubation-to-first mobilisation interval was 14.9 ± 9.3 hours. Among patients with documented mobilisation timing, 102 patients were mobilised within 6 hours, 96 within 24 hours, and 2 beyond 24 hours post-extubation. Earlier mobilisation was associated with lower postoperative pain scores. Patients mobilised within 6 hours demonstrated lower mean VAS scores at 6, 24, 48, and 72 hours compared to those mobilised at 24 hours. The mean discharge VAS and ODI scores were 3.0 and 18.2, respectively, with an average hospital stay of 4.3 ± 1.6 days. Higher analgesic requirements, including tramadol-containing combinations and rescue Butrum analgesia, were associated with relatively higher pain scores and delayed mobilisation. Earlier mobilisation was more commonly observed following minimally invasive lumbar procedures, whereas delayed mobilisation was associated with multilevel fusion procedures, greater blood loss, and longer operative duration.

Conclusion

The extubation-to-first mobilisation interval appears to be a simple and clinically meaningful parameter reflecting early postoperative recovery following major spine surgery. Earlier mobilisation was associated with lower postoperative pain scores and favourable recovery trends. Larger prospective multicentre studies with adjustment for potential confounders are required to determine whether earlier mobilisation independently influences postoperative outcomes.

## Introduction

Early mobilisation following major spine surgery is increasingly recognised as an essential aspect of enhanced recovery protocols, with growing evidence that timely ambulation can reduce postoperative morbidity and length of hospital stay and improve overall outcomes [1]. The conventional practice of prolonged bed rest after surgery has been linked to several adverse outcomes, including an increased risk of thromboembolic events, pneumonia, muscle atrophy, and overall physical deconditioning [2]. Early mobilisation is recommended to mitigate respiratory complications [3] and reduce the incidence of thromboembolic events while also contributing to improved functional recovery after major elective surgical procedures [4,5].

The Enhanced Recovery After Surgery (ERAS) concept is based on integrating a series of evidence-based perioperative and postoperative interventions aimed at minimising surgery-related physiological stress, thereby facilitating faster and improved patient recovery without increasing complication rates [6]. These protocols were conceptualised initially for colorectal surgery patients, but over the years, applications of ERAS protocols have widened in spine surgeries to include complex deformity corrections, cervical spine surgeries, and lumbar fusion surgeries [7-12]. Recent systematic reviews and meta-analyses continue to support ERAS pathways in spine surgery, demonstrating improvements in postoperative recovery, earlier mobilisation, reduced opioid consumption, shorter hospital stay, and lower healthcare costs without increasing postoperative complications. These contemporary findings further reinforce the importance of standardised perioperative care and early rehabilitation in patients undergoing major spine surgery.

One crucial yet underexplored factor influencing postoperative recovery is the interval between extubation and initiation of mobilisation, which may be affected by patient comorbidities, intraoperative factors, and perioperative management strategies. Although extubation timing and postoperative airway management have received particular attention in complex spine procedures [12], there is limited prospective data specifically evaluating how the extubation-to-mobilisation interval relates to recovery trajectories and postoperative complications in this population.​

This study aims to prospectively assess the interval from extubation to first mobilisation in patients undergoing major spine surgery, seeking to identify patterns, related perioperative factors, and associations with key clinical outcomes. By focusing on this critical transition, the results may inform best practices for perioperative management and early rehabilitation protocols in spine surgery.

## Materials and methods

Study design and setting

This was a prospective observational study conducted at Stavya Spine Hospital and Research Institute, Ahmedabad, following approval from the institutional ethics committee (approval number: SSHRI/CS/NS/TAT/80/12.25). This study evaluates when first mobilisation occurs (typically within 24-48 hours post-surgery) and its impact on pain, complication rates, and hospital stay. Data collection includes baseline demographics, hospital stay duration, and outcome assessments at discharge.

Study population

Adult patients aged 18-80 years undergoing major elective spine surgery were prospectively enrolled. Major spine surgery was defined as elective spinal procedures performed under general anaesthesia requiring postoperative inpatient admission and including instrumented fusion, deformity correction, revision surgery, tumour surgery, or complex decompression procedures. Minor spine procedures such as day-care microdiscectomy, vertebroplasty, kyphoplasty, or simple decompression not requiring inpatient postoperative rehabilitation were excluded.

Inclusion criteria

The inclusion criteria were as follows: patients aged 18-80 years undergoing planned major spine surgery (multilevel fusion, instrumentation, and complex decompression), ability to walk preoperatively, and an upper age limit of 80 years to reduce heterogeneity arising from extreme age-related frailty, multiple comorbidities, and markedly different postoperative rehabilitation potential.

Exclusion criteria

Those with preexisting immobility or severe neurological deficits, acute infection, malignancy, or trauma cases, and severe comorbidities (cardiac or respiratory) that preclude safe mobilisation were excluded.

Preoperative data

Preoperative data collected included demographic characteristics such as age, sex, and body mass index (BMI). Baseline clinical status was assessed using Visual Analog Scale (VAS) pain scores. Relevant comorbidities, including diabetes mellitus, smoking history, and cardiovascular disease, were also recorded to evaluate their potential influence on postoperative recovery and mobilisation outcomes.

Intraoperative data

Intraoperative data included the type of surgical procedure performed, operative duration, estimated blood loss, number of levels operated, and any intraoperative complications. Details regarding anaesthesia and perioperative medications were also recorded to assess their potential influence on postoperative recovery and mobilisation.

Postoperative data

Postoperative data included the interval from extubation to first mobilisation (out-of-bed ambulation), serial pain assessments using VAS scores at 6, 24, 48 and 72 hours, and daily analgesic consumption. Functional outcomes were evaluated using Oswestry Disability Index (ODI) and Visual Analog Scale (VAS) scores at discharge. Length of hospital stay and postoperative complications, including wound infection, deep vein thrombosis, reoperation, readmission, and neurological deficits, were also recorded.

Mobilisation protocol as per institutional ERAS/guideline

Postoperative day 0-1 involves sitting at bedside or chair with medical assessment for readiness. Day 1-2 requires first assisted ambulation (standing/walking) with documentation of timing (hours from extubation). Day 2 onward focuses on gradual increase in walking distance/frequency, functional exercises, and stair climbing as tolerated.

As this was a prospective observational study, patients were not assigned to predefined mobilisation groups. The timing of first mobilisation reflected routine postoperative clinical practice and depended on factors such as surgical complexity, hemodynamic stability, pain control, neurological status, and physiotherapist assessment. Consequently, the number of patients in each mobilisation category was determined by actual clinical recovery and was not expected to be equally distributed.

## Results

A total of 267 patients undergoing major spine surgery were prospectively evaluated. The mean extubation-to-first mobilisation interval was 14.9 ± 9.3 hours. Among patients with documented mobilisation timing, 102 patients were mobilised within 6 hours, 96 patients within 24 hours, and 2 patients beyond 24 hours post-extubation. The mean discharge VAS score was 3.0, mean ODI score was 18.2, and the average length of hospital stay was 4.3 ± 1.6 days (Table 1).

**Table 1:** Overall postoperative outcomes Values are presented as mean ± SD for continuous variables and number (%) for categorical variables. Mobilisation timing was available for 200 patients and was analysed on an available-case basis. VAS: Visual Analog Scale, ODI: Oswestry Disability Index, SD: standard deviation

Variable	Value
Total patients	267
Documented mobilisation timing	200
Mean extubation-to-first mobilisation interval (hours)	14.9 ± 9.3
Mobilised within 6 hours, number (%)	102 (51%)
Mobilised within 24 hours, number (%)	96 (48%)
Mobilised beyond 24 hours, number (%)	2 (1%)
Discharge VAS score	3.0 ± 0.9
Discharge ODI score	18.1 ± 5.9
Length of hospital stay (days)	4.3 ± 1.6

Overall, the incidence of postoperative complications was low during the study period. No major neurological deterioration or mortality was observed. A small number of patients experienced minor postoperative events that were managed conservatively without significantly affecting overall recovery. Owing to the low event rate, subgroup comparison according to mobilisation timing was not performed.

Association between mobilisation timing and postoperative outcomes

Earlier mobilisation was associated with significantly lower postoperative pain scores (Table 2). Patients mobilised within 6 hours demonstrated lower mean VAS scores at 6 hours (5.8 ± 0.7 versus 8.0 ± 1.0; U = 429.0, p < 0.001), 24 hours (4.4 ± 0.9 versus 5.7 ± 0.8; U = 1594.5, p < 0.001), 48 hours (3.8 ± 0.9 versus 4.8 ± 1.2; U = 2683.5, p < 0.001), and 72 hours (3.0 ± 0.9 versus 4.0 ± 1.1; U = 2336.0, p < 0.001) compared with patients mobilised at 24 hours.

**Table 2:** Association between mobilisation timing and postoperative outcomes Values are presented as mean ± SD. Patients were stratified according to mobilisation within 6 hours and 24 hours following extubation. Continuous variables were compared using the independent samples t-test or Mann-Whitney U test, as appropriate. Lower VAS scores indicate less postoperative pain. Statistical significance was defined as p < 0.05. VAS: Visual Analog Scale, SD: standard deviation

Outcome	≤6 hour mobilisation	24 hour mobilisation	Test	Test statistic	p-value
VAS at 6 hours	5.8 ± 0.7	8.0 ± 1.0	Mann-Whitney U	U = 429.0	<0.001
VAS at 24 hours	4.4 ± 0.9	5.7 ± 0.8	Mann-Whitney U	U = 1594.5	<0.001
VAS at 48 hours	3.8 ± 0.9	4.8 ± 1.2	Mann-Whitney U	U = 2683.5	<0.001
VAS at 72 hours	3.0 ± 0.9	4.0 ± 1.1	Mann-Whitney U	U = 2336.0	<0.001

Analgesic utilisation

With regard to analgesic requirements, the majority of patients received analgesics according to the World Health Organisation (WHO) analgesic ladder. Dynapar (diclofenac) formed the backbone of multimodal analgesia and was administered in 128 patients, most commonly as twice-daily dosing for approximately three days. Intravenous/oral paracetamol preparations (Febrinil 650/Neomol/Cafipar) were frequently used as an adjunct analgesic. Higher analgesic requirement, defined as the need for tramadol-containing combinations (Ramcet/Ultracet) were administered in 70 patients overall, while Butrum rescue analgesia was required in only 4 patients (Table 3).

**Table 3:** Analgesic utilisation Values are presented as frequencies (number). Dynapar (diclofenac) and paracetamol (Febrinil/Neomol/Cafepar) formed the baseline multimodal analgesic regimen. Tramadol-containing combinations and Butrum were considered escalation analgesic therapies for breakthrough or inadequately controlled postoperative pain.

Analgesic	Number	Definition/remarks
Dynapar (diclofenac), paracetamol (Febrinil/Neomol/Cafepar)	128	Baseline non-opioid multimodal analgesia
Tramadol-containing combinations (Ramcet/Ultracet)	70	Higher analgesic requirement
Butrum rescue analgesia	4	Rescue analgesia

Analgesic requirement and postoperative outcomes

Patients requiring higher analgesic support demonstrated slightly higher postoperative pain scores and marginally delayed mobilisation compared with those managed using standard analgesia alone (Table 4). Butrum use was limited to a very small subset of patients with comparatively higher immediate postoperative pain and slower early mobilisation, suggesting that only a minority required escalation to stronger rescue analgesia. Mean VAS scores in the higher-analgesic group were 7.1 ± 1.4 versus 6.8 ± 1.4 at 6 hours (U = 5163.0, p = 0.146), 5.2 ± 1.1 versus 5.0 ± 1.1 at 24 hours (U = 5147.0, p = 0.155), 4.5 ± 1.2 versus 4.2 ± 1.1 at 48 hours (U = 5020.5, p = 0.275), and 3.5 ± 1.2 versus 3.5 ± 1.1 at 72 hours (U = 4518.5, p = 0.884). Mobilisation time was also slightly longer in the higher-analgesic group (15.8 ± 9.8 versus 14.3 ± 9.0 hours; t = 1.06, p = 0.289). However, none of these differences reached statistical significance.

**Table 4:** Analgesic requirement and postoperative outcomes Values are presented as mean ± SD. Higher analgesic requirement was defined as the use of tramadol-containing combinations (Ramcet/Ultracet) and/or Butrum rescue analgesia. Continuous variables were compared using the independent samples t-test or Mann-Whitney U test, as appropriate. Statistical significance was defined as p < 0.05. VAS: Visual Analog Scale, SD: standard deviation

Outcome	Higher analgesic requirement*	Standard analgesia	Test	Test statistic	p-value
VAS at 6 hours	7.1 ± 1.4	6.8 ± 1.4	Mann-Whitney U	U = 5163.0	0.146
VAS at 24 hours	5.2 ± 1.1	5.0 ± 1.1	Mann-Whitney U	U = 5147.0	0.155
VAS at 48 hours	4.5 ± 1.2	4.2 ± 1.1	Mann-Whitney U	U = 5020.5	0.275
VAS at 72 hours	3.5 ± 1.2	3.5 ± 1.1	Mann-Whitney U	U = 4518.5	0.884
Mobilisation time (hours)	15.8 ± 9.8	14.3 ± 9.0	Welch t-test	t = 1.06	0.289

Surgical factors affecting mobilisation

Procedure type also influenced mobilisation timing and pain trajectories. Earlier mobilisation was more commonly achieved following minimally invasive lumbar procedures such as single-level minimally invasive spine surgery-transforaminal lumbar interbody fusion (MISS-TLIF) and limited decompression surgeries, whereas delayed mobilisation was more frequently observed after multilevel fusion procedures and longer cervical/thoracolumbar reconstructions associated with greater blood loss and operative duration. Patients mobilised within 6 hours also had significantly lower intraoperative blood loss (122.0 ± 76.9 mL versus 213.7 ± 154.1 mL; t = -5.15, p < 0.001) and shorter operative duration (112.3 ± 33.2 minutes versus 133.4 ± 41.1 minutes; t = -3.88, p < 0.001) than those mobilised at 24 hours (Table 5).

**Table 5:** Surgical factors associated with mobilisation timing An independent samples t-test was used to compare surgical factors between patients mobilised within 6 hours and those mobilised within 24 hours. A p-value <0.05 was considered statistically significant. Both blood loss and operative duration were significantly lower in the early mobilisation group.

Surgical factor	≤6 hour mobilisation	24 hour mobilisation	Test	Test statistic	p-value
Blood loss (mL)	122.0 ± 76.9	213.7 ± 154.1	Welch t-test	t = -5.15	<0.001
Operative duration (minutes)	112.3 ± 33.2	133.4 ± 41.1	Welch t-test	t = -3.88	<0.001

## Discussion

Early mobilisation is a cornerstone of modern perioperative care, particularly within the framework of Enhanced Recovery After Surgery (ERAS) protocols [6]. The present prospective study evaluates a relatively underexplored yet clinically critical parameter (the interval between extubation and first mobilisation) and its relationship with postoperative recovery in patients undergoing major spine surgery. By focusing on this transition phase, our study attempts to bridge a gap between anaesthetic recovery and functional rehabilitation. The findings of this study suggest that earlier mobilisation following extubation is associated with favourable postoperative outcomes, including improved pain trajectories, reduced complication rates, and shorter hospital stay. These observations reinforce the concept that the immediate postoperative period represents a modifiable window of recovery, where timely intervention can influence downstream clinical outcomes. Unlike traditional metrics that evaluate mobilisation based on postoperative day counts, our study introduces a time-sensitive parameter (hours from extubation), which provides a more precise and clinically actionable measure of early recovery dynamics.

Comparison with existing literature

The benefits of early mobilisation in surgical patients are well documented. Previous studies across surgical disciplines have demonstrated that delayed ambulation is associated with increased risks of venous thromboembolism, pulmonary complications, and prolonged hospitalisation [13-15]. ERAS-based spine surgery protocols have similarly emphasised early mobilisation as a key determinant of improved outcomes [10,15-20]. However, most existing spine literature defines early mobilisation broadly (e.g., mobilisation within 24-48 hours), without examining the precise timing from extubation to mobilisation. Our study adds to the literature by suggesting that even within this early window, shorter extubation-to-mobilisation intervals may confer incremental benefits. More recent evidence has further strengthened the role of ERAS in spine surgery. A large systematic review and meta-analysis involving over 15,000 patients demonstrated that ERAS pathways are associated with earlier mobilisation, reduced postoperative pain, shorter hospital stay, lower opioid consumption, and fewer perioperative complications while maintaining patient safety. These findings support the integration of structured mobilisation protocols within contemporary spine ERAS pathways [21,22]. This aligns with physiological principles: early ambulation improves pulmonary mechanics, venous return, and muscle activation, thereby reducing complications such as atelectasis, deep vein thrombosis, and deconditioning.

Physiological and clinical implications

The interval between extubation and first mobilisation is influenced by a complex interaction of patient-specific, surgical, anaesthetic, and institutional factors, which can act as confounders or effect modifiers and contribute to heterogeneous outcomes. Patient-related variables such as age, baseline functional status, pain tolerance, and comorbidities (e.g., cardiopulmonary disease, obesity, and diabetes) may affect both physiological recovery and readiness for mobilisation. Intraoperative factors, including duration of surgery, extent of tissue dissection, number of levels operated, and blood loss, can influence postoperative fatigue, pain, and hemodynamic stability, thereby delaying mobilisation [6,20]. The observed association between earlier mobilisation and improved postoperative recovery may, in part, reflect differences in surgical invasiveness. Patients undergoing minimally invasive procedures generally experienced shorter operative duration, lower blood loss, and less postoperative pain, which may have facilitated earlier mobilisation. Although operative duration and blood loss were compared between groups, residual confounding cannot be excluded. Future studies incorporating multivariable regression analyses adjusting for procedure type and surgical complexity are needed to determine whether the extubation-to-mobilisation interval independently predicts postoperative recovery.

Anaesthetic considerations play a particularly important role. The choice of anaesthetic agents, depth and duration of anaesthesia, use of long-acting opioids, and adequacy of reversal can all impact the speed of recovery from sedation and the patient’s ability to safely ambulate. Similarly, variations in postoperative pain management strategies, particularly the use of multimodal analgesia versus opioid-heavy regimens, can significantly influence early mobilisation, either by enabling comfortable movement or, conversely, by causing sedation and delayed responsiveness. In addition, institutional practices and perioperative protocols contribute to variability. Differences in ERAS implementation, physiotherapy availability, staffing patterns, and surgeon-specific preferences regarding mobilisation timing can lead to inconsistent mobilisation practices even within similar patient populations. Psychological factors, including patient motivation, fear of pain or reinjury, and perioperative counselling, may further influence the willingness to mobilise early [20].

Taken together, these factors can cause significant variation in the extubation-to-mobilisation interval between different patients and clinical settings. This highlights the importance of interpreting mobilisation timing within the overall clinical context and emphasises the need for standardised protocols and adjusted analyses when assessing its effect on postoperative outcomes. Within ERAS pathways, minimally invasive surgery facilitates earlier mobilisation through reduced tissue trauma, lower postoperative pain, and faster functional recovery. The observed earlier mobilisation among MISS patients in the present study is consistent with these principles.

Several limitations should be acknowledged. First, this was a single-centre observational study, which may limit the generalisability of the findings. Second, mobilisation timing was unavailable for a proportion of patients, introducing the possibility of selection bias. Third, all analyses were unadjusted, and important confounders such as age, BMI, comorbidities, surgical invasiveness, operative duration, blood loss, and anaesthetic factors may have influenced the observed associations. Fourth, although postoperative complications were prospectively recorded, the incidence was low, and no statistically meaningful comparison between mobilisation groups could be performed; therefore, complication outcomes were not emphasised. Finally, the observational design precludes causal inference, and the extubation-to-first mobilisation interval should be interpreted as an indicator of postoperative recovery rather than an independent determinant of improved clinical outcomes.

## Conclusions

The extubation-to-first mobilisation interval appears to be a practical marker of early postoperative recovery following major spine surgery. Earlier mobilisation was associated with lower postoperative pain scores and favourable recovery trends. However, because of the observational design and potential confounding factors, these findings should be interpreted as associations rather than evidence of causality. Larger multicentre prospective studies incorporating multivariable analyses are required to determine whether earlier mobilisation independently improves postoperative outcomes.
